# Comparison of two labor induction regimens with intravaginal misoprostol 25 μg and adverse perinatal outcomes

**DOI:** 10.1590/1806-9282.20240286

**Published:** 2024-08-30

**Authors:** Marcela Beraldo Santiago, Talita Beraldo Santiago, Samuel Machado Oliveira, João Victor Jacomele Caldas, Edward Araujo, Alberto Borges Peixoto

**Affiliations:** 1 Universidade de Uberaba, Mário Palmério University Hospital, Gynecology and Obstetrics Service – Uberaba (MG), Brazil.; 2 Universidade Federal de São Paulo, Escola Paulista de Medicina, Department of Obstetrics – São Paulo (SP), Brazil.; 3 Universidade Municipal de São Caetano do Sul, Discipline of Women Health – São Caetano do Sul (SP), Brazil.; 4 Universidade Federal do Triângulo Mineiro, Department of Obstetrics and Gynecology – Uberaba (MG), Brazil.

**Keywords:** Labor induction, Misoprostol, Outcomes assessment

## Abstract

**OBJECTIVE:**

The aim of the study was to compare two labor induction regimens (4 and 6 h), to determine predictors of successful labor induction with intravaginal misoprostol 25 μg tablets, and to evaluate the association with adverse perinatal outcomes.

**METHODS:**

This was a retrospective cohort study that included singleton pregnancies undergoing induction of labor with an intravaginal misoprostol 25 μg tablet between 37 and 42 weeks of gestation. The pregnant women were divided into two groups: Group 1—intravaginal misoprostol 25 μg every 4 h and Group 2—intravaginal misoprostol 25 μg every 6 h.

**RESULTS:**

Pregnant women were divided into Group 1 (n=289) and Group 2 (n=278). Group 1 had a higher median number of intravaginal misoprostol 25 μg tablets (3.0 vs. 2.0 tablets, p<0.001), a lower prevalence of postpartum hemorrhage (7.6 vs. 32.7%, p<0.001), and a higher need for oxytocin (odds ratio [OR]: 2.1, 95%CI: 1.47–2.98, p<0.001) than Group 2. Models including intravaginal misoprostol 25 μg tablets every 4 and 6 h [x^2^(1)=23.7, OR: 4.35, p<0.0001], parity [x^2^(3)=39.4, OR: 0.59, p=0.031], and Bishop’s score [x^2^(4)=10.8, OR: 0.77, p=0.019] were the best predictors of failure of labor induction. A statistically significant difference between groups was observed between the use of the first intravaginal misoprostol 25 μg tablet at the beginning (Breslow p<0.001) and the end of the active labor phase (Long Hank p=0.002).

**CONCLUSION:**

Pregnant women who used intravaginal misoprostol 25 μg every 4 h had a longer time from the labor induction to the beginning of the active phase of labor and higher rates of adverse perinatal outcomes than women who used intravaginal misoprostol 25 μg every 6 h.

## INTRODUCTION

Labor induction differs from labor management in that it artificially stimulates effective uterine contractions and cervical ripening before they manifest spontaneously^1^. It is a widely used procedure in clinics and hospitals, with several indications focusing on pregnant women and fetal safety in the presence of comorbidities such as premature rupture of membranes (PROM) and post-term pregnancies^2,3^.

Prostaglandins (PGE1 or PGE2) activate collagenase and promote dissociation of the collagen fibers of the cervix to allow passage of the fetus without causing dissolution, allowing the cervix to later return to its pre-pregnancy state^4^. Prostaglandins have been shown to increase the rate of labor after 24 h of induction, reducing the need for oxytocin administration and cesarean section rates^5^. Misoprostol is a synthetic analog of PGE1 and is useful in obstetrics for its uterotonic and cervical softening properties and can be administered by oral or vaginal route^6^. Currently, misoprostol is used to induce labor at a dose of 25 μg every 4 to 6 h^7^. The bioavailability of misoprostol by vaginal and rectal routes is higher than oral route because they do not have a first hepatic passage^7^. In addition, the oral route requires doses three to four times higher than the vaginal route and has a higher incidence of gastrointestinal effects, particularly nausea and vomiting^8^.

Misoprostol 25 μg vaginally in 6-h doses reduces the incidence of prolonged labor and does not increase the risk of tachysystole, meconium passage, fetal distress, and neonatal hypoxia. However, it is known that the most effective method of inducing labor is misoprostol 25 μg vaginally in 4-h doses, with a higher risk of uterine hyperstimulation^8^.

Failed induction has been described in several ways in the literature in both observational and randomized trials^10^. Definitions have included failed vaginal delivery^11^, failed initiation of active labor^12,13^, and failed labor after a certain number of ripening agents^14^. In some trials, no definition was provided in the protocol for failed induction^15,16^. Due to this lack of standardization, even among randomized controlled trials, it is not surprising that the term failed induction has an unclear meaning^10^. A Bishop’s score<6 within 4–6 h after the last intravaginal misoprostol 25 μg tablet may be considered a failure to induce labor, depending on the dose used^9^.

The objective of this study was to compare two labor induction regimens (4 and 6 h), determine predictors of successful induction of labor with intravaginal misoprostol 25 μg tablet, and evaluate the association with adverse perinatal outcomes.

## METHODS

This was a retrospective cohort study conducted in the Obstetrics Sector of Mário Palmério University Hospital, Uberaba, MG, Brazil, from March 2014 to November 2022, through research in medical records. The participants were divided into two groups: Group 1: pregnant women undergoing labor induction with intravaginal misoprostol 25 μg every 4 h and Group 2: pregnant women undergoing labor induction with intravaginal misoprostol 25 μg every 6 h. This study was approved by the Ethics Committee of the University of Uberaba (CAAE: 65887022.8.0000.5145), and the consent form was dispensed as it was a retrospective study.

We included singleton pregnancies undergoing induction of labor with intravaginal misoprostol 25 μg tablet between 37 and 42 weeks of gestation, with fetuses in cephalic presentation, gestational age calculated from the date of last menstrual period and confirmed by first-trimester obstetric ultrasound, absence of chromosomal disorders and structural abnormalities, and Bishop’s score ≤6.

According to the protocol of our service, patients with a Bishop score ≤6 start induction of labor with an intravaginal misoprostol 25 μg tablet. There was no randomization in the selection of misoprostol use during the induction of labor. The chosen dosage follows the institutional protocol and the need for adjustments due to the COVID-19 pandemic. Between March 2014 and January 2020, the institutional induction protocol was to use misoprostol 25 μg every 6 h. Between January 2020 and November 2022, the institutional protocol was modified in an attempt to reduce induction time and subsequent length of stay by changing the dosage to misoprostol 25 μg every 4 h.

At the end of every 4- or 6-h post-misoprostol period, the patient’s uterine cervix was re-evaluated through vaginal touch, and a new Bishop’s score was done. If the Bishop’s score>6, or if the pregnant woman had rhythmic uterine contractions, an intravenous oxytocin continuous infusion was started. In cases where a combination of the two methods was used, a 6-h interval was respected between the insertion of the intravaginal misoprostol 25 μg tablet and the beginning of the oxytocin infusion.

The presence of regular uterine contractions, cervical effacement of at least 80%, and cervical dilatation of 4–6 cm was considered the active phase of labor^17^. Failure of induction was defined as the absence of at least two painful, regular contractions in a 10-min evaluation and no progress in cervical effacement or dilation at the end of the 200 μg misoprostol dose.

The variables analyzed were maternal age, parity, gestational age, PROM, Bishop’s score, time from the beginning of labor induction and active phase, tachysystole, hasty labor, number of intravaginal misoprostol tablets, failure of labor induction, APGAR scores at the first and fifth minute, neonatal death, neonatal intensive care unit admission, and postpartum hemorrhage (PPH).

We considered PPH when there was a blood loss of 500 mL or more after vaginal delivery or 1,000 mL or more after cesarean section associated with a shock index (heart rate/systolic blood pressure ratio) of 0.9 or more, according to the “Zero Maternal Death from Postpartum Hemorrhage”^18^. We considered hasty labor when the fetal dilation, descent, and expulsion occurred in less than 4 h. Tachysystole was considered if there were >5 contractions in 10 min, averaged over a 30-min window^19^.

To calculate the sample volume, the GPower 3.1 program was used. Using an effect size of 0.25, a power of 80%, a confidence interval of 95%, and a significance level of 0.05 (probability of error of 5%), a sample size of 199 participants in the group who used misoprostol at a dosage every 4 h and 199 participants in the group who used misoprostol at a dosage every 6 h will be required to evaluate the effect of the dosage on the time between the beginning of the induction process and the active phase of labor. Using an effect size of 0.25, a power of 80%, a confidence interval of 95%, and a significance level of 0.05 (probability of error of 5%), a sample size of 80 participants in the group who used misoprostol at a dosage every 4 h and 80 participants in the group that used misoprostol at a dosage every 6 h will be required to evaluate the association of the studied group with adverse perinatal events. Using an odds ratio of 1.3, a power of 80%, a confidence interval of 95%, and a significance level of 0.05 (probability of error of 5%), a total sample of 567 participants will be necessary to evaluate the best predictors of induction failure in the two groups studied.

Data were collected in an Excel 2007 spreadsheet (Microsoft Corp., Redmond, WA, USA) and analyzed using SPSS version 20.0 (SPSS Inc., Chicago, IL, USA) and Prisma GraphPad version 7.0 (GraphPad Software, San Diego, CA, USA). The D’Agostino and Pearson normality tests were used to analyze whether the values had a Gaussian distribution. Non-parametric distribution variables were presented as medians and minimum and maximum values. The Mann-Whitney test was used to compare variables between groups. Categorical variables were described by absolute and percentage frequencies and presented in tables. The chi-square test was used to evaluate the association between the type of labor induction and the categorical variables. Binary logistic regression was initially used to calculate the odds ratio (OR) of the successful induction of labor. Subsequently, stepwise forward binary logistic regression was used to evaluate the best predictors of the successful induction of labor using the variables that showed a significant difference between the groups (intravaginal misoprostol 25 μg tablet every 4 h, intravaginal misoprostol 25 μg tablet every 6 h, number of pregnancies, parity, and Bishop’s score). The significance level for all tests was p<0.05.

## RESULTS

**Figure 1:**
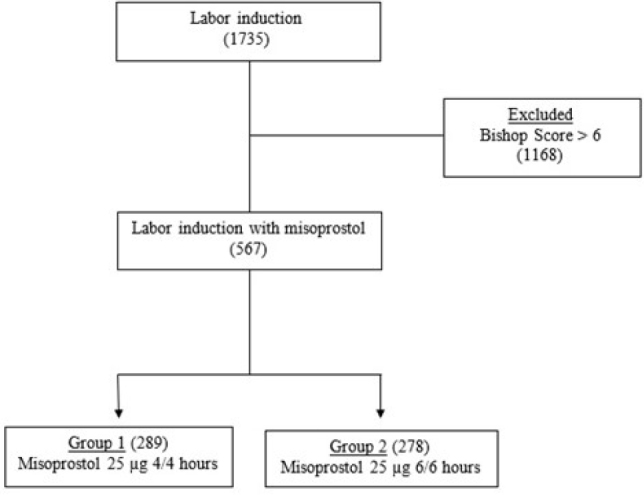
Flowchart of the included cases in the study.

From March 2014 to November 2022, 1,735 patients underwent labor induction. It excluded 1,168 patients who underwent labor induction with oxytocin due to a Bishop score>6. For final statistical analyses, 567 patients who underwent labor induction with intravaginal misoprostol were evaluated. The included patients were divided into two groups according to the dosage: Group 1—intravaginal misoprostol 25 μg tablet every 4 h (289) and Group 2—intravaginal misoprostol 25 μg tablet every 6 h (278) (Figure 1).

Group 1 pregnant women had higher parity (1.0 vs. 0.0, p<0.001), number of previous vaginal deliveries (1.0 vs. 0.0, p<0.001), and Bishop’s score (4.0 vs. 3.0, p<0.001) than Group 2. Group 1 pregnant women had a lower APGAR score at the first minute (8.0 vs. 9.0, p<0.001) and APGAR score at the fifth minute (8.0 vs. 9.0, p<0.001) than Group 2 (Table 1).

**Table 1:** Clinical characteristics of the study population.

	Group 1 (289)	Group 2 (278)	Statistics	p-value
Maternal age (years)	23.0 (15.0–43.0)	23.0 (14.0–45.0)	38.9	0.535^∫^
Number of previous pregnancies	1.0 (1.0–9.0)	1.0 (1.0–11.0)	39.5	0.715^∫^
Number of previous deliveries	1.0 (0.0–8.0)	0.0 (0.0–8.0)	28.6	<0.001^∫^
Number of vaginal deliveries	1.0 (0.0–10.0)	0.0 (0.0–10.0)	33.3	<0.001^∫^
Number of cesarean sections	0.0 (0.0–2.0)	0.0 (0.0–1.0)	33.4	0.815^∫^
Gestational age (weeks)	40.0 (37.0–42.1)	40.0 (37.0–43.0)	39.4	0.691^∫^
Bishop’s score	4.0 (1.0–8.0)	3.0 (1.0–7.0)	32.5	<0.001^∫^
Premature rupture of ovular membranes	23.2% (67/289)	27.2% (77/278)	1.52	0.217^ƒ^
Type of delivery			0.41	0.520^ƒ^
Vaginal	64.7% (187/289)	67.3% (187/278)		
Cesarean section	35.3% (102/289)	32.7% (91/278)		
Birth weight (g)	3,230 (2,070–4,685)	3,272 (2,180–4,230)	37.5	0.179^∫^
APGAR score at the first minute	8.0 (1.0–10.0)	9.0 (2.0–10.0)	25.8	<0.001^∫^
APGAR score at the fifth minute	8.0 (5.0–10.0)	9.0 (6.0–10.0)	31.3	<0.001^∫^

Group 1: intravaginal misoprostol 25 μg every 4 h; Group 2: intravaginal misoprostol 25 μg every 6 h; Mann-Whitney

∫: median (minimum–maximum); chi-square

ƒ: percentage (n/N). p<0.05.

Group 1 pregnant women had a higher median number of intravaginal misoprostol 25 μg tablets (3.0 vs. 2.0 tablets, p<0.001) than Group 2. Group 1 pregnant women had a lower prevalence of postpartum hemorrhage (7.6 vs. 32.7%, p<0.001) than Group 2. Group 1 pregnant women have a higher likelihood of failure of labor induction (OR: 3.50, 95%CI 2.05–5.93, p<0.001), need for oxytocin (OR: 2.1, 95%CI 1.47–2.98, p<0.001), and tachysystole (OR: infinity, 95%CI 17.8–infinity) than Group 2. Group 1 pregnant women have a lower risk of postpartum hemorrhage than Group 2 (OR: 0.16, 95%CI 0.10–0.27, p<0.001) (Table 2).

**Table 2:** Characteristics of labor induction and adverse perinatal outcomes with induction using intravaginal misoprostol 25 μg every 4 and 6 h.

	Group 1 (289)	Group 2 (278)	Statistics	p-value
Number of intravaginal misoprostol tablets	3.0 (1.0–8.0)	2.0 (1.0–8.0)	32.0	<0.001^∫^
Oxytocin use	46.4% (134/289)	29.1% (81/278)	17.0	<0.001^ƒ^
Failure of labor induction	20.4 % (59/289)	6.8% (19/278)	22.0	<0.001^ƒ^
APGAR score at first minute <7	6.2% (22/353)	8.5% (18/212)	1.03	0.311^ƒ^
Admission of neonatal intensive care unit	1.7% (5/289)	1.8% (5/278)	0.003	0.951^ƒ^
Hasty labor	19.0% (55/289)	0.0% (0/278)	58.6	<0.001^ƒ^
Tachysystole	1.0% (3/289)	0.4% (1/278)	0.931	0.335^ƒ^
Postpartum hemorrhage	7.6% (22/289)	32.7% (91/278)	56.0	<0.001^ƒ^
Neonatal death within 48 h	0.0% (0/289)	0.0% (0/278)	–	–

Group 1: intravaginal misoprostol 25 μg every 4 h; Group 2: intravaginal misoprostol 25 μg every 6 h; Mann-Whitney

∫: median (minimum–maximum); chi-square

ƒ: percentage (n/N). p<0.05.

Group 1 pregnant women had a longer median maximum time between the beginning of misoprostol use and the beginning of active labor compared to Group 2 (9.5 vs. 6.0 h, respectively). A statistically significant difference between groups was observed between the use of the first intravaginal misoprostol 25 μg tablet at the beginning (Breslow p<0.001) and the end of the active labor phase (Long Hank p=0.002).

Considering all cases included in the study, a stepwise forward binary logistic regression model was created using intravaginal misoprostol 25 μg tablet every 4 and 6 h, the number of pregnancies, parity, and Bishop’s score to assess the best predictors of failure of labor. The number of pregnancies [x^2^(2)=34.7, OR: 0.97, p=0.899, R^2^ Nagelkerke=0.109] lost its ability to predict failure of labor induction after the inclusion of parity in the model. The model including intravaginal misoprostol 25 μg tablet every 4 and 6 h [x^2^(1)=23.7, OR: 4.35, p<0.0001, R^2^ Nagelkerke=0.075], parity [x^2^(3)=39.4, OR: 0.59, p=0.031, R^2^ Nagelkerke=0.123], and Bishop’s score [x^2^(4)=10.8, OR: 0.77, p=0.019, R^2^ Nagelkerke=0.142] were the best predictors of failure of labor induction (Table 3).

**Table 3:** Risk of failure of labor induction in pregnant women undergoing induction with intravaginal misoprostol 25 μg every 4 and 6 h, number of pregnancies, number of deliveries, and Bishop’s score as predictors.

	OR	95%CI	X^2^	R^2^ (Nagelkerke)	p-value
Intravaginal misoprostol 25 μg every 4 and 6 h	4.35	2.38–7.93	23.7	0.075	<0.0001
Number of pregnancies	0.97	2.56–8.42	34.7	0.109	0.899
Number of deliveries	0.59	0.36–0.95	39.4	0.123	0.031
Bishop’s score	0.77	0.62–0.95	10.8	0.142	0.019

Stepwise forward binary logistic regression. p<0.05.

## DISCUSSION

Rates of labor induction have increased significantly over the past few decades^20^. According to a study in Denmark, the rate of induction of labor in term pregnancies increased from 12.4 to 25.1%, a growth of 108%^21^. Given the robust and significant statistics, it is crucial that the specialist dealing with the pregnancy-puerperium cycle has a solid knowledge of the subject.

The main pharmacological method to induce labor in unfavorable cervical conditions includes prostaglandins, which catalyze the collagen network of the uterine cervix, facilitating its fading and dilation. In Brazil, the main drug used is PGE1, due to its lower cost and the lack of driving, whose main representative is misoprostol. The dose used in the main obstetric services is 25 μg vaginally every 6 h, as recommended by the International Federation of Obstetrics and Gynecology^22^. Other regimens are also accepted in the literature, mainly regarding the higher frequency of doses every 4 h^23,24^. Serious adverse perinatal outcomes associated with the use of misoprostol are similar to those of other prostaglandins and include uterine tachysystole, with its potential fetal and maternal effects, and meconium staining of the cerebrospinal fluid. It is generally agreed that it is a potent uterotonic and should not be used in women with a previous cesarean section because it increases the risk of uterine rupture^23^. A randomized controlled trial in 124 women using several different single doses (25, 50, 100, and 200 μg) resulted in more vaginal deliveries at 12 and 24 h, more tachysystole, and less need for oxytocin with each increasing dose^25^. A double-blind, randomized, controlled trial of 374 women (>36 weeks, Bishop score ≤4) administered either 100 or 200 μg of a single intravaginal misoprostol had similar results, with the higher dose resulting in significantly more women achieving vaginal delivery within 24 h (24 vs. 36%), a shorter time from labor induction to delivery (1,181 vs. 1,744 min), and less use of oxytocin (49 vs. 71%), but an increased rate of tachysystole (41 vs. 19.5%)^26^. In our sample, the highest total dose and fractionation at shorter intervals were associated with greater failure of labor induction and hasty labor but were not associated with tachysystole, probably because of the dose-dependent rather than time-dependent effect. Ozbasli et al.^27^ compared three groups: spontaneous labor, labor induction with a single intravaginal misoprostol 25 μg tablet, and multiple intravaginal misoprostol 25 μg tablets. The time from the last dose of misoprostol to delivery was statistically shorter for pregnant women who received a single dose of misoprostol. The use of oxytocin was higher in primiparous pregnant women who received multiple doses. There were no differences between the groups with regard to fetal distress.

A randomized controlled trial in 204 women comparing oral misoprostol 25 μg with intravaginal misoprostol 50 μg vaginal given every 4 h for up to four doses found that the oral route had lower incidence of tachysystole (2.2 vs. 5.4%) and lower cesarean section rates (19.4 vs. 32.4%), but no difference in induction of labor or side effects (nausea, vomiting, chills, or diarrhea) than the intravaginal route^28^. In our study, the frequency of tachysystole was not significantly different between the groups, probably because the final dose in cases of failure of labor induction was similar. In a systematic review of five randomized controlled trials, Sanchez-Ramos et al.^29^ found that tachysystole and hyperstimulation syndrome appeared to be less common in pregnant women who received intravaginal misoprostol 25 μg than in those who received 50 μg. There was no difference in adverse perinatal outcomes between the groups. The 50 μg intravaginal misoprostol group was associated with a shorter interval to vaginal delivery, a greater proportion of deliveries within 24 h, and less need for oxytocin.

In our study, pregnant women who used intravaginal misoprostol 25 μg every 4 h had a longer time to the beginning of the active phase of labor. In a prospective cohort study, Porras Lucena et al.^30^ evaluated 300 pregnant women undergoing labor induction, being: Group 1 (n=150)—intravaginal misoprostol 50 μg followed by 25 μg every 4 h and Group 2 (n=150)—initial dose of intravaginal misoprostol 25 μg followed by the same dose every 4 h. Group 1 showed a reduction in time to delivery and the need for oxytocin compared to Group 2. No differences in perinatal outcomes were observed between the groups.

## CONCLUSION

In summary, pregnant women who used intravaginal misoprostol 25 μg every 4 h had a longer time from the labor induction to the beginning of the active phase of labor and higher rates of adverse perinatal outcomes than intravaginal misoprostol 25 μg every 6 h.
